# Amlodipine Ameliorates Ischemia-Induced Neovascularization in Diabetic Rats through Endothelial Progenitor Cell Mobilization

**DOI:** 10.1155/2016/3182764

**Published:** 2016-05-08

**Authors:** Jiayin Sun, Jun Xie, Lina Kang, Albert Ferro, Li Dong, Biao Xu

**Affiliations:** ^1^Department of Cardiology, Beijing Anzhen Hospital, Capital Medical University, Beijing 100029, China; ^2^Department of Cardiology, Affiliated Drum Tower Hospital, Nanjing University Medical School, 321 Zhongshan Road, Nanjing 210008, China; ^3^Cardiovascular Division, British Heart Foundation Centre of Research Excellence, King's College London, 150 Stamford Street, London SE1 9NH, UK; ^4^Department of Cardiology, Hospital of Nanjing Municipal Authorities, Nanjing 210008, China

## Abstract

*Objectives*. We investigated whether amlodipine could improve angiogenic responses in a diabetic rat model of acute myocardial infarction (AMI) through improving bone marrow endothelial progenitor cell (EPC) mobilization, in the same way as angiotensin converting enzyme inhibitors.* Methods*. After induction of AMI by coronary artery ligation, diabetic rats were randomly assigned to receive perindopril (2 mgkg^−1^ day^−1^), amlodipine (2.5 mgkg^−1^ day^−1^), or vehicle by gavage (*n* = 20 per group). Circulating EPC counts before ligation and on days 1, 3, 5, 7, 14, and 28 after AMI were measured in each group. Microvessel density, cardiac function, and cardiac remodeling were assessed 4 weeks after treatment. The signaling pathway related to EPC mobilization was also measured.* Results*. Circulating EPC count in amlodipine- and perindopril-treated rats peaked at day 7, to an obvious higher level than the control group peak which was reached earlier (at day 5). Rats treated with amlodipine showed improved postischemia neovascularization and cardiac function, together with reduced cardiac remodeling, decreased interstitial fibrosis, and cardiomyocyte apoptosis. Amlodipine treatment also increased cardiac SDF-1/CXCR4 expression and gave rise to activation of VEGF/Akt/eNOS signaling in bone marrow.* Conclusions*. Amlodipine promotes neovascularization by improving EPC mobilization from bone marrow in diabetic rats after AMI, and activation of VEGF/Akt/eNOS signaling may in part contribute to this.

## 1. Introduction

Diabetes mellitus (DM) is an independent predictor of adverse outcomes in patients with acute myocardial infarction (AMI) [1, 2]. This condition is associated with impairment of ischemia-induced neovascularization and of collateral vessel growth [3, 4], which leads to larger infarct area, more severe left ventricular (LV) remodeling, and more impaired LV function [5]. Accumulating evidence suggests that DM is accompanied by endothelial progenitor cell (EPC) dysfunction [6, 7], which has emerged as an important concept in explaining dysfunctional neovascularization [8, 9]. Indeed, in line with this, we have previously shown that EPC mobilization from the bone marrow in response to AMI is impaired and associated with poor prognosis in patients with type 2 DM [10]. These considerations raise the possibility that ameliorating ischemia-induced neovascularization in the context of diabetes by increasing the level of circulating EPCs mobilized from the bone marrow may improve prognosis.

Amlodipine is a dihydropyridine calcium-channel blocker that is widely used for the treatment of hypertension. Clinical and experimental studies have shown that amlodipine has some blood pressure- (BP-) independent effects, including reducing risk of adverse cardiovascular events in patients with coronary heart disease (CHD) without hypertension, attenuating LDL-induced endothelial dysfunction, and alleviating ischemia-reperfusion-induced heart injury [11–13]. Moreover, amlodipine prevents LV remodeling and improves cardiac function after AMI [14, 15], though the underlying mechanism is unclear. We have previously reported that amlodipine, unlike other calcium-channel blockers, can to some degree inhibit serum angiotensin converting enzyme (ACE) activity and increase nitric oxide (NO) release from vascular endothelial cells, similar to ACE inhibitors [16]. ACE inhibitors, which are widely used in patients with cardiovascular disease, have been demonstrated to exert beneficial effects on EPC biology [17–19]. They can enhance the release of NO through activating the protein kinase B (Akt)/endothelial NO synthase (eNOS) pathway [20, 21], and NO in turn has an essential role in mobilization of EPCs, an event which is required for myocardial neovascularization and survival after AMI [22, 23]. Based on these considerations, we wished to examine whether amlodipine may influence CHD development and recovery through NO-mediated EPC mobilization, in the same way as ACE inhibitors.

In this study, we investigated whether amlodipine can improve the angiogenic responses in a diabetic rat model following ligation of left anterior descending (LAD) coronary artery, through activation of bone marrow EPC mobilization. We compared amlodipine responses to those to the ACE inhibitor perindopril as a positive control. We firstly examined the therapeutic potential of amlodipine for ameliorating the impaired angiogenesis in ischemic myocardium and then secondly investigated the role of the VEGF/Akt/eNOS/MMP-9 pathway in the bone marrow in relation to EPC mobilization.

## 2. Materials and Methods

### 2.1. Diabetic AMI Model

The animal studies presented here were approved by the Nanjing University Scientific and Animal Ethics Committee (approval number 20080125) and were in compliance with the Chinese national regulations on the use of experimental animals. SPF Sprague-Dawley rats (male, 200–250 g) were used to induce experimental diabetes [24, 25]. After four-week high-fat diet (40% fat), blood samples were obtained to analyze insulin, cholesterol, and triglyceride concentrations, following which rats were injected with a low dose of streptozotocin (STZ, 30 mg/kg) intraperitoneally. Oral glucose tolerance test (OGTT) and insulin tolerance test (ITT) were conducted five days after STZ injection, and insulin sensitivity index (ISI) was calculated as

(1) $$\begin{array}{c} ISI={\ln\left(\text{fasting blood glucose}\times \text{fasting insulin}\right)}^{-1}. \end{array}$$

Rats with >10 mmolL^−1^ fasting blood glucose (FBG) or >16.7 mmolL^−1^ postprandial blood glucose (PBG) were identified as diabetic (*n* = 80). Rats on normal chow diet with FBG < 7 mmolL^−1^ and PBG < 10 mmolL^−1^ (*n* = 20) served as nondiabetic for blood lipid, OGTT, and ITT measurements.

Diabetic rats received high-fat diet and water* ad libitum *for another four weeks after establishment of diabetes and were anaesthetized by intraperitoneal administration of a mixture of ketamine hydrochloride (50 mgkg^−1^) and diazepam (5 mgkg^−1^). Rats were then endotracheally intubated and mechanically ventilated (Jiangxi Teli, China) with supplemental oxygen, following which thoracotomy was performed and myocardial infarction was induced by surgical ligation of the LAD 4 mm below the tip of the left atrial appendage. Successful ligation was verified by color change and electrocardiography. After recovery and three hours after LAD ligation, rats were randomly assigned to receive perindopril (2 mgkg^−1^ day^−1^; *n* = 20) or amlodipine (2.5 mgkg^−1^ day^−1^; *n* = 20), each dissolved in 0.5% sodium carboxymethyl cellulose (MCT-Na), or 1 mL 0.5% MCT-Na (control; *n* = 20), by daily gavage for four weeks. During this period, heart rate (HR) and systolic BP (SBP) in the conscious state were determined once a week using a tail-cuff pressure analysis system (Softron BP-98A, Softron Biotechnology Ltd.), which included a programmable routine of cuff inflation and deflation, analysis and assignment of pulse rate and blood pressure, and recording of data electronically. Rats' tails were passed through a cuff, and BP was measured by determining the cuff pressure at which blood flow to the tail was eliminated.

### 2.2. Evaluation of Circulating EPC Number and Plasma VEGF

Circulating EPC counts of rats in each group (*n* = 15) were determined by flow cytometry before LAD ligation and on days 1, 3, 5, 7, 14, and 28 thereafter. Bone-marrow-derived early-stage EPCs were characterized as CD45^−/low+^ mononuclear cells (MNCs) (FITC-conjugated mouse anti-rat CD45, Invitrogen) coexpressing CD133 (rabbit anti-rat CD133, Abcam; goat anti-rabbit IgG conjugated with PE-Cy5.5, Invitrogen) as well as the endothelial specific antigen VEGF-2 (biotin-conjugated mouse anti-rat VEGF receptor 2, Novus Biologicals; Streptavidin-biotin APC, eBioscience). Cells were analyzed by quantitative 3-color flow cytometry using a fluorescence-activated cell sorter (FACSCanto, Becton Dickinson), and CD45^−/low+^CD133^+^VEGF-2^+^ EPC count was expressed as number per 10^6^ MNCs. At the same time points as EPC measurements were performed, plasma samples were separated and stored at −80°C until analysis. Plasma concentrations of VEGF (*n* = 5 in each group) were measured by enzyme-linked immunosorbent assay (Abcam) according to the manufacturer's instructions.

### 2.3. Assessment of Cardiac Function by Echocardiography

Before LAD ligation and four weeks after myocardial infarction, transthoracic echocardiography (SONOS 5500, Philips Medical Systems, Best, Netherlands; *n* = 10 in each group) was performed by a blinded investigator. Two-dimensional and M-mode images were examined from parasternal short-axis and long-axis view, from which left ventricular end-diastolic diameter (LVEDd), left ventricular end-systolic diameter (LVEDs), percentage of LV systolic ejection fraction (%EF), and percentage of LV fractional shortening (%FS) were calculated. All measurements were taken over at least three consecutive pulsation cycles and averaged.

### 2.4. Quantitation of BNP mRNA

BNP mRNA was quantitated by real-time polymerase chain reaction. After echocardiography, hearts were arrested in diastole by intravenous injection of 2 mL 10% KCl and then rapidly excised and snap-frozen in liquid nitrogen (*n* = 8 in each group) or placed in 10% buffered formalin (*n* = 4 in each group). Total RNA (*n* = 4 in each group) was isolated from the border zone around the infarction sites of frozen hearts using TRIzol reagent (Invitrogen) and stored at −80°C until analysis. After dissolving in DEPC-treated water, the RNA concentration of each sample was detected using Eppendorf BioPhotometer Plus, and 500 ng total RNA was reverse-transcribed into cDNA using the PrimeScript RT Master Mix (Takara Bio). Real-time polymerase chain reaction (RT-PCR) was performed with SYBR Premix Ex Taq*™* (Takara Bio) in a 7500 Real-Time PCR system (Applied Biosystems, CA, USA). The primers for BNP and GAPDH were as follows:

*BNP*

 f: CAGCTCTCAAAGGACCAAGG
 r: GCCCAAAGCAGCTTGAACTA

*GAPDH*

 f: GGGCTCTCTGCTCCTCCCTGTT
 r: CAGGCGTCCGATACGGCCAA

The relative gene expression for each sample was determined using the formula 2^−ΔΔCt^ to reflect target gene expression normalized to GAPDH levels.

### 2.5. Histological Studies and Detection of Collagen Volume Fraction

Four hearts per group obtained from sacrificed rats were used to make paraffin sections. Masson's trichrome staining was performed for cardiac remodeling and fibrosis detection. Infarct wall thickness, LV diameter, and LV wall thickness were analyzed by Image-Pro Plus (version 5.0), as was analysis of scar size, expressed as a percentage of total LV. Each of the stained slices was also used to measure collagen volume fraction (CVF). When magnified 200-fold, the areas positively stained by aniline blue were delineated by Image-Pro Plus (version 5.0) and expressed as a percentage of the total reference area.

### 2.6. TUNEL Assay

We detected apoptosis using the Dead End Fluorometric TUNEL System (Promega), according to the manufacturer's instructions. Four frozen hearts per group were sliced into 5 *μ*m thick sections using a freezing microtome. Frozen sections were counterstained with mouse monoclonal *α*-sarcomeric actin antibody (1 : 75, Abcam), and Alexa Fluor 594 rabbit anti-mouse antibody (1 : 1000, Invitrogen) was applied as secondary antibody; cell nuclei were stained with 4′,6-Diamidino-2-Phenylindole, Dilactate (DAPI; Sigma). The stained sections were mounted and analyzed with a Fluoview 1000 confocal microscope (Olympus, Japan). We counted 6 random 400x fields and the number of TUNEL-positive cardiomyocyte nuclei was manually determined. The total number of nuclei (exhibited as DAPI-positive signals) was automatically calculated using Image-Pro Plus (version 5.0).

### 2.7. Detection of Small Artery and Microvessel Density

Frozen sections were used for immunofluorescence by labelling with antibodies against CD31 (1 : 1000, BD Biosciences) or against von Willebrand factor (vWF, 1 : 800, Abcam) to observe microvessel density. Anti-*α*-smooth muscle actin (SMA) antibody (1 : 500, Abcam) was applied for examining small artery density. The number of vessels was counted from at least 6 randomly selected fields in the border zone of infarction under a magnification of 400x for capillaries and 100x for small arteries.

### 2.8. Quantitation of Stromal Cell-Derived Factor-1 (SDF-1) and CXCR4 mRNA

Total RNA (*n* = 4 in each group) was isolated from the border zone around the infarction sites of frozen hearts using TRIzol reagent (Invitrogen). RT-PCR was performed as mentioned above. The primers for SDF-1/CXCR4 and GAPDH were as follows: 

*SDF-1*

 f: GCTCTGCATCAGTGACGGTAAG
 r: TGGCGACATGGCTCTCAAA

*CXCR4*

 f: ATCATCTCCAAGCTGTCACACTCC
 r: GTGATGGAGATCCACTTGTGCAC

*GAPDH*

 f: GGGCTCTCTGCTCCTCCCTGTT
 r: CAGGCGTCCGATACGGCCAA

The relative gene expression for each sample was determined using the formula 2^−ΔΔCt^ to reflect target gene expression normalized to GAPDH levels.

### 2.9. Western Blot Analysis and Gelatin Zymography

For western blot analyses, bone marrow extracts were prepared and western blotting was performed as previously described [26]. Akt (1 : 2000, BD Biosciences), phospho-Akt (1 : 1000, BD Biosciences), eNOS (1 : 1000, BD Biosciences), phospho-eNOS (1 : 500, BD Biosciences), and matrix metalloproteinase- (MMP-) 9 (1 : 15,000, Abcam) were quantified in the bone marrow tissue to assess changes after AMI. Expression of proteins and phosphorylation levels was normalized to *β*-actin (1 : 2000, Santa Cruz) and to baseline expression. MMP-9 activity was assessed by gelatin zymography and quantification of bands was performed using image software Quantity One (Bio-Rad). Human recombinant MMP-9 (Chemicon) was used as positive control.

### 2.10. Statistical Analysis

All continuous variables were expressed as mean ± SD. Comparisons between groups were performed by unpaired Student's *t*-test or one-way ANOVA with Dunnett's posttest as appropriate. In all cases, statistical significance was defined as *p* < 0.05 (two-tailed). All analyses were performed using SPSS 17.0 software (SPSS, Inc.).

## 3. Results

### 3.1. Animal Model of Diabetes

**Figure 1:**
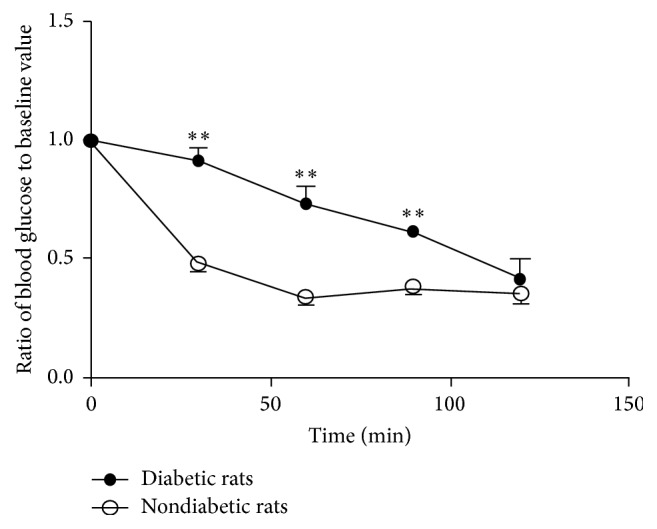
Insulin tolerance test results for diabetic and nondiabetic rats. *n* = 4 per group. ^*∗∗*^ *p* < 0.01.

High-fat diet coupled with low dose STZ injection was used to induce diabetes in rats. Plasma cholesterol, triglycerides, and fasting blood insulin levels were increased after four weeks of high-fat diet (prior to STZ injection) (Table 1). FBG and PBG were both raised after STZ injection, whilst ITT showed a delayed reduction of plasma glucose in diabetic rats (Figure 1), and ISI was also reduced, all indicating establishment of diabetes and insulin resistance. In response to amlodipine and perindopril, HR and SBP were unchanged compared to untreated rats.

### 3.2. Amlodipine Enhances EPC Mobilization in Diabetic Animals after AMI

**Figure 2:**
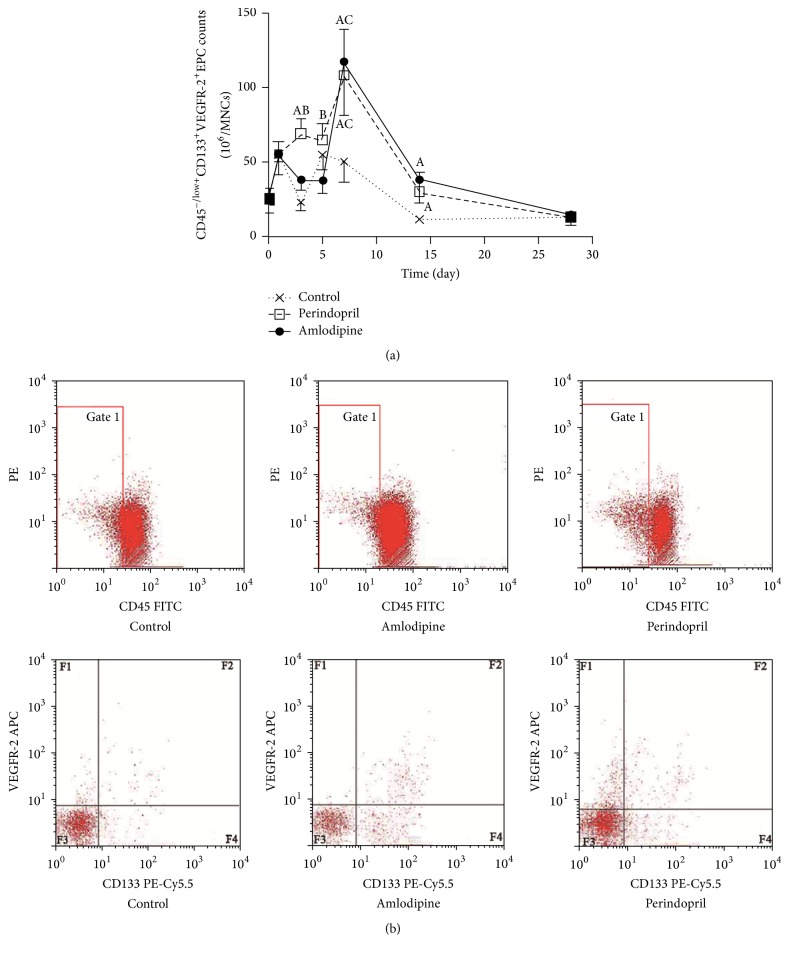
Effects of amlodipine and perindopril on EPC mobilization in diabetic rats after AMI. (a) CD45^−/low+^CD133^+^VEGFR-2^+^ cell numbers as a function of time in rats after AMI treated with amlodipine, perindopril, or vehicle (control) (*n* = 15 per group). (b) Two-step analysis by flow cytometry to quantify CD45^−/low+^CD133^+^VEGFR-2^+^ EPCs at peak (amlodipine and perindopril group at day 7, control group at day 5). CD45^−/low+^ EPC subset analysis was conducted on the Gate 1 region using fluorescein isothiocyanate- (FITC-) labelled antibody to CD45. The percentage of CD133^+^VEGFR-2^+^ cells was analyzed in the F2 region by two-color flow cytometry using a combination of phycoerythrin-Cy5.5- (PE-Cy5.5-) labelled monoclonal antibody to CD133 and allophycocyanin- (APC-) labelled monoclonal antibody directed to VEGFR-2. A: *p* < 0.05 versus control group at the same time point, B: *p* < 0.05 versus amlodipine group at peak, and C: *p* < 0.05 versus control group at peak.

Figure 2(a) shows circulating EPC counts in diabetic rats following AMI, expressed as CD45^−/low+^CD133^+^VEGFR-2^+^ cells/10^6^ MNCs. All groups of rats had similar EPC levels at baseline. In rats on no-drug therapy, circulating EPCs exhibited two peaks: at days 1 and 5 after AMI, the latter peak being maintained up to day 7 and followed by a rapid decline. In the perindopril-treated group, EPC counts rose similarly at day 1 but continued to rise thereafter reaching a maximum at day 7, which was of much greater magnitude than the maximum seen in controls (108 ± 30/10^6^ MNCs versus 55 ± 10/10^6^ MNCs, *p* < 0.05). On the other hand, in rats receiving amlodipine, the initial EPC profile was similar to that seen in untreated animals, with a peak at day 1 to levels similar to control animals, declining at day 3 to levels much lower than those seen in the perindopril group (EPC count 38 ± 7/10^6^ MNCs in amlodipine group versus 69 ± 10/10^6^ MNCs in perindopril group at day 3, *p* < 0.05); however, after day 5, there was a steep rise in circulating EPCs, so that by day 7 EPC levels in amlodipine-treated rats were similar to those seen in the perindopril-treated group. In both perindopril- and amlodipine-treated animals, the higher circulating EPC count lasted up to day 14, at which point EPC levels in both of these groups were of the order of 3-fold greater than untreated rats.  Figure 2(b) illustrates typical flow cytometry analyses to quantify circulating CD45^−/low+^CD133^+^VEGFR-2^+^ EPCs in rats at peak (control group on day 5; perindopril and amlodipine groups on day 7).

### 3.3. Amlodipine Improves Cardiac Function after AMI in Diabetic Rats

**Figure 3:**
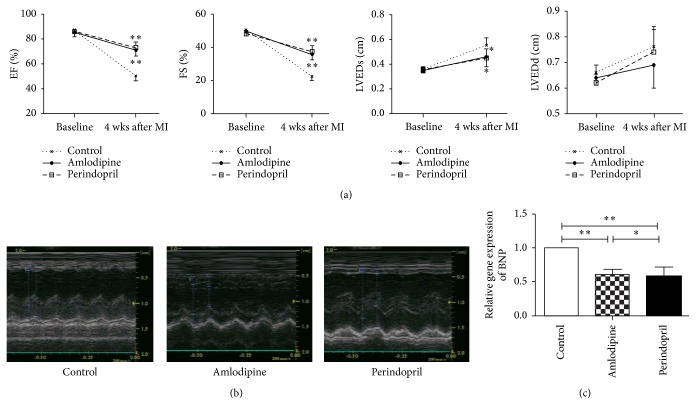
Effects of amlodipine and perindopril on cardiac function after AMI in diabetic rats. (a) Shown are ejection fraction (EF), fractional shortening (FS), left ventricular end-systolic diameter (LVEDs), and left ventricular end-diastolic diameter (LVEDd), as assessed echocardiographically. (b) Typical M-mode echocardiographic traces in rats from each of the three groups. (c) BNP gene expression as assessed by real-time PCR. *n* = 10 per group assessed by echocardiography and *n* = 4 per group for real-time PCR assessments.^*∗*^ *p* < 0.05 versus control group and ^*∗∗*^ *p* < 0.01 versus control group.

Echocardiography was used to assess cardiac function four weeks after AMI. Compared with control animals, diabetic rats treated with amlodipine exhibited increased systolic function, as manifested by improved % FS and EF and reduced LVEDs, very similar to the effect of perindopril (Figure 3(a)). Typical M-mode images in rats from each of the three groups are shown in Figure 3(b).

### 3.4. Amlodipine Decreases BNP mRNA Level in the Border Zone of Infarction in Diabetic Rats

In order to characterize the effect of amlodipine and perindopril on cardiac systolic function and cardiac remodeling, we examined expression of BNP by RT-PCR. Rats treated with either amlodipine or perindopril exhibited markedly reduced levels of BNP mRNA in the border area around ischemic regions of the LV, indicative of improved cardiac function (Figure 3(c)).

### 3.5. Amlodipine Improves Cardiac Remodeling and Inhibits Cardiomyocytes Apoptosis in Diabetic Rats after AMI

**Figure 4:**
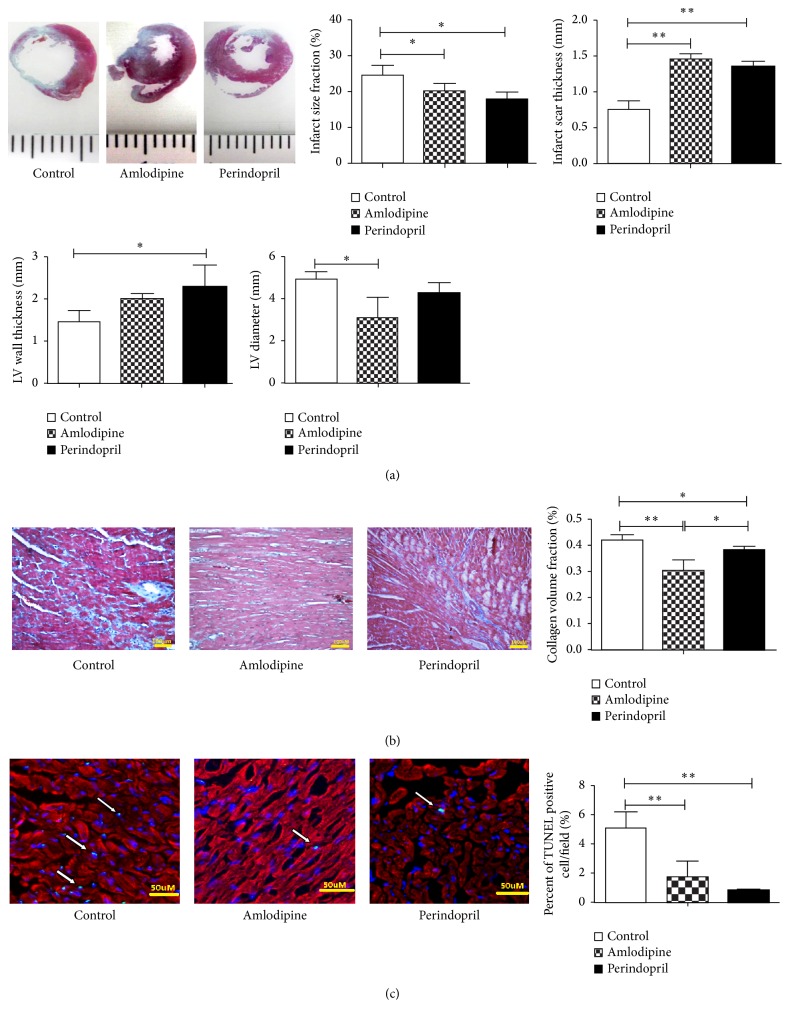
Effects of amlodipine and perindopril on left ventricular structure in diabetic rats after AMI. (a) Masson's trichrome staining of myocardial sections after four weeks of treatment, with analysis of infarct size, infarct scar thickness, and cardiac remodeling by quantification of left ventricular (LV) diameter and wall thickness. (b) Masson's trichrome staining showing myocardial fibrosis in the three groups. (c) Cardiomyocyte apoptosis as revealed by triple-staining with TUNEL (green, arrows), anti-*α*-sarcomeric actin antibody (red), and DAPI (blue) in the infarct border zone. *n* = 4 per group. ^*∗*^ *p* < 0.05 and ^*∗∗*^ *p* < 0.01. Scale bar = 100 *μ*m for (b) and scale bar = 50 *μ*m for (c).

Consistent with the observed improvement in cardiac function, we also found that both amlodipine and perindopril inhibit cardiac remodeling after AMI. As shown in Figure 4(a), histological analysis revealed a reduced infarct size together with a thicker infarct scar in amlodipine- or perindopril-treated rats. LV diameter in the amlodipine group decreased to almost half that in control group, reflecting attenuation of LV dilation by amlodipine; this same effect was not seen in perindopril-treated animals, or at any rate any reduction in LV diameter with perindopril was less in magnitude and did not reach statistical significance. Moreover, LV wall thickness was increased by perindopril, but not significantly by amlodipine. Microscopically, interstitial fibrosis, determined from CVF on Masson's trichrome staining, was decreased by treatment with either perindopril or amlodipine for one month and was in fact greater in the amlodipine than in the perindopril group (Figure 4(b)). On TUNEL analysis, the percentage of TUNEL-positive cardiomyocytes was decreased to a similar degree by either amlodipine or perindopril, the observed reduction being of the order 60–70% compared to the control group (Figure 4(c)).

### 3.6. Amlodipine Increases after Ischemia Neovascularization in Diabetic Rats following AMI

**Figure 5:**
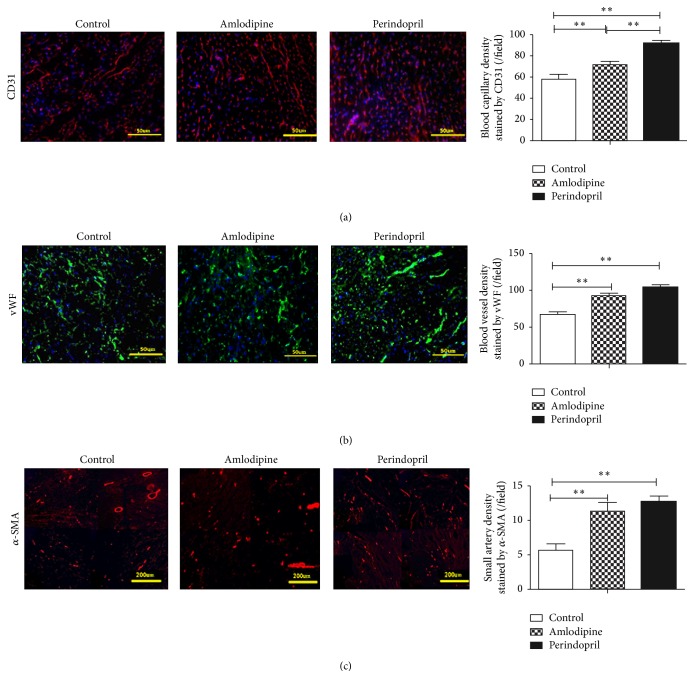
Effects of amlodipine and perindopril on neovascularization in diabetic rats after AMI. (a) Capillary formation in infarct border zone as stained by CD31 (red). (b) Blood vessel formation in heart as stained by vWF (green). (c) Small artery formation in heart as stained by *α*-SMA (red); each picture was comprised of four fields. Nuclei were stained by DAPI (blue). Blood vessel density was expressed as the number of capillaries per field (*n* = 4 in each group). ^*∗*^ *p* < 0.05 and ^*∗∗*^ *p* < 0.01. Scale bar = 50 *μ*m for (a and b) and scale bar = 200 *μ*m for (c).

In response to perindopril, an increase in capillary density was noted by CD31 and vWF staining; additionally, the number of small arteries, assessed by staining for *α*-SMA, was also increased to a level almost 1.5-fold greater than untreated rats. Amlodipine treatment gave rise to a similar increase in small arteries and a lesser increase in capillaries, as compared to perindopril (Figure 5).

### 3.7. Amlodipine Augments Cardiac SDF-1 and CXCR4 Expression in Diabetic Rats after AMI

**Figure 6:**
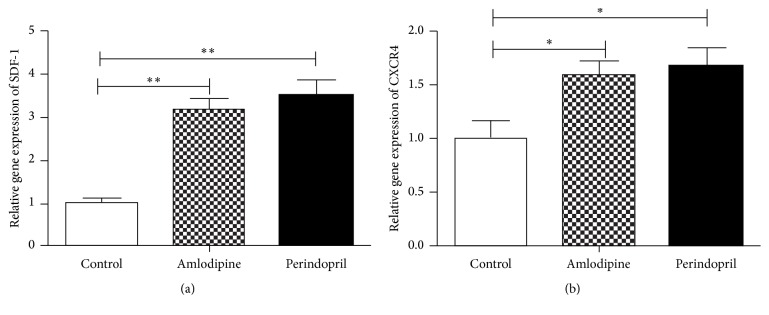
Effects of amlodipine and perindopril on SDF-1 (a) and CXCR4 (b) mRNA in diabetic rats after AMI. ^*∗*^ *p* < 0.05 and ^*∗∗*^ *p* < 0.01.

SDF-1/CXCR4 pathway has been demonstrated to play a crucial role in directing EPC homing to ischemic tissue [27]. We have previously indicated that ACE inhibitor perindopril would increase plasma SDF-1 expression in diabetic patients after myocardial infraction [19]. To determine whether either SDF-1 or CXCR4 expression may be altered by amlodipine, we finally examined mRNA levels in the border zone around the infarction sites of hearts. The experiment revealed that both SDF-1 and CXCR4 mRNA were markedly increased in perindopril- or amlodipine-treated rats, while there was no obvious difference between the two treated groups (Figure 6).

### 3.8. Amlodipine Increases Plasma VEGF and Upregulates Bone Marrow Expression of Phospho-Akt, Phospho-eNOS, and MMP-9 in Diabetic Rats after AMI

**Figure 7:**
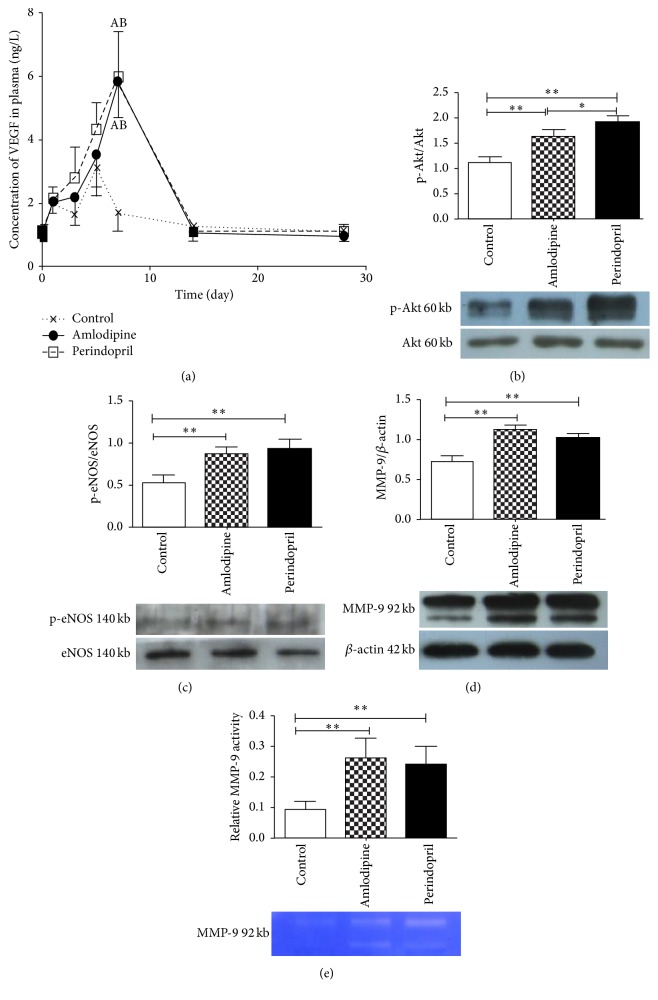
Effects of amlodipine and perindopril on signaling pathways involved in bone marrow EPC mobilization, in diabetic rats after AMI. Effects are shown on (a) plasma VEGF level and on bone marrow expression of (b) phospho-Akt as a ratio to total Akt, (c) phospho-eNOS as a ratio to total eNOS, and (d) MMP-9 as a ratio to the housekeeping protein *β*-actin. (e) Bone marrow MMP-9 activity as measured by gelatin zymography. *n* = 5 per group; A: *p* < 0.05, compared with control group at the same time point; B: *p* < 0.05, compared with control group at peak. ^*∗*^ *p* < 0.05 and ^*∗∗*^ *p* < 0.01; VEGF: vascular endothelial growth factor; p-Akt: phosphorylated protein kinase B; p-eNOS: phosphorylated endothelial nitric oxide synthase; and MMP-9: matrix metalloproteinase-9.

We wished to explore whether EPC mobilization by amlodipine and perindopril in this model was mediated through VEGF/Akt/eNOS/MMP-9 signaling. We therefore measured plasma VEGF level at the same time points as determination of EPC numbers. The kinetic of VEGF in the control group was similar to those of EPC, with a slight elevation at day 1 after AMI and reaching a maximum at day 5 (3.10 ± 0.83 ng/L). In both drug-treated groups, the level of VEGF increased rapidly between days 3 and 5, reaching maximum at day 7 to a level which was much higher than that in untreated animals (5.83 ± 1.13 ng/L in amlodipine group versus 1.12 ± 0.24 ng/L in control group at day 7, *p* < 0.05; 5.89 ± 1.42 ng/L in perindopril group versus 1.12 ± 0.24 ng/L in control group at day 7, *p* < 0.05) (Figure 7(a)).

Western blotting revealed that bone marrow expression of both phospho-Akt and phospho-eNOS was markedly increased after treatment with either drug for 7 days to levels 1.5- to 2-fold greater than in untreated animals (Figures 7(b)-7(c)), with no change in total Akt or eNOS expression. At the same time point, both amlodipine and perindopril also increased bone marrow expression (Figure 7(d)) and activity (Figure 7(e)) of MMP-9.

## 4. Discussion

Patients with diabetes exhibit a worse clinical outcome after AMI, as manifest both by more severe ischemia and by poorer prognosis [28]. This is believed to result from dysfunctional angiogenesis due to defective EPC mobilization in response to ischemic injury [10]. Our purpose was to examine the effects of amlodipine, which in addition to its calcium-channel blocking activity also appears to inhibit ACE [16], on EPC mobilization and neovascularization, as well as on cardiac remodeling and function. We found in diabetic rats with myocardial infarction induced by LAD ligation that, like perindopril, amlodipine caused a marked increase in circulating EPCs lasting up to 14 days after AMI, and this led to increased formation of capillaries and small arteries. On histological analysis, increased survival of ischemic myocardium and decreased tissue fibrosis were observed in hearts of amlodipine-treated rats.

In addition, echocardiography and measurement of cardiac BNP mRNA level were used to document the beneficial effect of amlodipine on cardiac function in this model. On echocardiography, amlodipine treatment—just as with perindopril treatment—improved % FS and LVEDs, indicating better contractile function, as also confirmed by increased EF. Moreover, B-type natriuretic peptide (BNP) has been recognized as a useful marker for predicting acute and chronic left ventricular dysfunction. The BNP gene encodes the 108 amino acid prohormone proBNP, which is split into BNP hormone and NT-proBNP in the circulation. Many studies indicated that there was a significant reverse relation between BNP and EF and a significant relationship between BNP and cardiac remodeling in the follow-up [29, 30]. By the way, NT-proBNP was found to be a better predictive indicator for infarct size and left ventricle function after AMI in the follow-up [31, 32]. We found that level of BNP mRNA was decreased in the area around infarction after amlodipine treatment, which indicated improved cardiac function and remodeling. Besides this, improvement of LV diameter was observed histologically in amlodipine-treated rats, even though this did not reach significance for LVEDd on echocardiography, possibly due to insufficient power to detect this in the present study. The improvement in LV function in the amlodipine-treated group could be attributed to a combination of decreased infarct size, thicker infarct scar, and reduced myocardial fibrosis. Furthermore, it is likely that increased mobilization of EPCs from bone marrow accompanied by increased neovascularization in the infarcted heart played a pivotal role in all of these changes.

Formation of collateral vessels in response to vascular occlusive disease determines to a large extent the severity of residual tissue ischemia. Emerging data indicate that such neovascularization does not exclusively rely on sprouting from local vessels, but also involves circulating EPCs derived from bone marrow [33, 34]. Moreover, NO is considered to be crucial for EPC mobilization, and ACE inhibition is known to increase EPCs by increasing NO levels [21]. Although amlodipine, which can suppress plasma ACE activity, has been reported to increase endothelial NO release [34, 35] and to be beneficial in preserving the coronary microvasculature as well as inducing angiogenesis of capillaries and small vessels [36, 37], few studies have examined whether its effect on angiogenesis is related to EPC mobilization. The only study in the literature to do so was that by Fukao et al., who investigated the effect of 12 weeks' treatment with amlodipine on hematopoietic progenitor cells in nondiabetic patients with essential hypertension: however their study proved negative [38]. In our present study, we found that the impairment in ischemia-induced mobilization of bone-marrow-derived EPCs in diabetic rats was improved by amlodipine. Increased circulating EPCs can contribute to ongoing endothelial repair by providing a circulating pool of cells that can home into injured segments of artery or can replace dysfunctional endothelial cells [28, 39]. Moreover EPCs, once mobilized into the peripheral circulation, localize to infarcted myocardium and are involved in new vessel formation [40], which then improve blood supply in the ischemic border zone, thereby resulting in decreased area of infarction, improved LV function, and reduced LV remodeling after AMI [41].

Several lines of evidence indicate that circulating EPCs originate from bone marrow and that this involves a complex interaction between cytokines and other molecules, including hypoxia inducible factor-1 (HIF-1*α*), VEGF, fibroblast growth factor-2 (FGF-2), and stromal cell-derived factor-1 (SDF-1). These factors may be involved in both regulation of EPC mobilization and their homing to ischemic myocardium. SDF-1 signaling through CXCR4 has been demonstrated to augment ischemic neovascularization not only through enhancing EPC recruitment in ischemic tissues [27], but also through the creation of a permissive microenvironment that facilitates the actions of VEGF [42]. Furthermore, ACE inhibitor perindopril has been shown to increase SDF-1 and CXCR4 expression [43]. In our study, we found that, as perindopril, amlodipine could also augment SDF-1 and CXCR4 expression in ischemic heart, which would contribute to EPC homing and VEGF action. Since the VEGF/Akt/eNOS signaling pathway as well as MMP-9 has been shown to play an important part in mobilizing EPCs [44, 45] and amlodipine has been identified to increase expression of both VEGF and MMP-9 [34, 46], we examined the effect of amlodipine on the components of this pathway. We found that VEGF level was increased in peripheral blood in response to amlodipine or perindopril treatment, as was the expression in bone marrow of phospho-Akt, phospho-eNOS, and MMP-9. These data indicate that activation of VEGF/Akt/eNOS signaling pathway, thereby giving rise to increase NO production, may be involved in mobilizing EPC from bone marrow and may contribute, at least in part, to neovascularization after amlodipine therapy. It should be noted however that EPC mobilization occurred during the first few days after AMI in treated or untreated diabetic rats, whilst plasma VEGF levels were still relatively low, and this may reflect the participation of many other mediators such as HIF-1*α* and SDF-1*α* in EPC mobilization.

In this study, we also observed prevention of cardiomyocyte apoptosis in the hearts of amlodipine-treated rats. Since apoptosis of myocardial cells is an important component underlying the reduction of cardiac function in the setting of AMI [47], inhibiting apoptosis is an important potential therapeutic option to attenuate postinfarction cardiac remodeling and dysfunction. Perindopril has been shown to modulate endothelial apoptosis and renewal via effects on EPCs in patients with acute coronary syndromes [48]; and amlodipine has been demonstrated to inhibit apoptosis in doxorubicin-induced neonatal rat cardiac myocytes and granulation tissue cell after infarction [15, 49]. We believe that the effects of amlodipine on cardiomyocyte apoptosis in the border zone of infarction in our model are due to improvement in angiogenesis consequent on EPC mobilization, and the antiapoptotic effect observed may rather relate to improved cardiac remodeling and function.

## 5. Conclusions

In conclusion, we have demonstrated that administration of amlodipine to diabetic rats after AMI promotes neovascularization by activating EPC mobilization from bone marrow, and this may be explained at least in part by activation of VEGF/Akt/eNOS and MMP-9 signaling. Amlodipine therapy also prevents cardiac remodeling and ameliorates cardiac function due to improved angiogenesis after infarction in this model. The therapeutic potential of our findings as they relate to diabetic patients with AMI remains to be determined.

## Figures and Tables

**Table 1:** Characteristics of diabetic and nondiabetic rats.

	Nondiabetics	Diabetics	*p*
*n*	20	80	
Weight (g)			
Week 0	233.15 ± 7.71	228.98 ± 8.35	0.25
Week 4	361.19 ± 24.38	431.14 ± 27.42	<0.01
Week 8	452.6 ± 21.9	448.9 ± 37.8	0.67
Glucose (mM)			
FBG week 5	4.13 ± 0.43	16.14 ± 2.61	<0.01
PBG week 5	10.56 ± 1.24	30.42 ± 3.31	<0.01
Triglycerides (mM)	0.762 ± 0.165	2.369 ± 1.497	0.002
Cholesterol (mM)	1.54 ± 0.30	2.21 ± 0.62	0.003
Fasting insulin (*μ*IU/mL)	34.304 ± 4.822	73.402 ± 9.734	0.004
ISI	−4.931 ± 0.736	−7.622 ± 0.763	<0.01

All plasma determinations were performed in the fasting state. Lipid and fasting insulin were measured at week 4, whilst fasting and postprandial blood glucose were measured 5 days after STZ (week 5). FBG: fasting blood glucose, PBG: postprandial blood glucose, and ISI: insulin sensitivity index.

## Additional Points

Since diabetes is an independent predictor of adverse outcomes after AMI which is associated with impaired neovascularization, ameliorating ischemia-induced neovascularization in the context of diabetes may improve prognosis. We found that amlodipine could improve the angiogenic responses through endothelial progenitor cell mobilization in diabetes and ameliorate cardiac function after AMI, and activation of VEGF/Akt/eNOS signaling may in part contribute to this. The results of this study represent a novel principle potentially involved in cardiovascular repair after AMI in diabetic patients.
